# Psychological Functioning and Disease-Related Quality of Life in Pediatric Patients With an Implantable Cardioverter Defibrillator

**DOI:** 10.1007/s00246-012-0175-1

**Published:** 2012-02-08

**Authors:** H. M. Koopman, C. M. J. Vrijmoet-Wiersma, J. N. D. Langius, F. van den Heuvel, S. A. Clur, C. A. Blank, N. A. Blom, A. D. J. ten Harkel

**Affiliations:** 1 Department of Medical Psychology, Leiden University Medical Center, Leiden, The Netherlands; 2 Department of Pediatric Cardiology, University Medical Center, Groningen, The Netherlands; 3 Department of Pediatric Cardiology, Academic Medical Center, Amsterdam, The Netherlands; 4 Department of Pediatric Cardiology, University Medical Center, Utrecht, The Netherlands; 5 Department of Pediatric Cardiology, Leiden University Medical Center, Leiden, The Netherlands

**Keywords:** Pediatric patients, Implantable cardioverter defibrillator, Disease-related quality of life, Psychological functioning

## Abstract

The objective of this multicenter study was to evaluate psychological functioning and disease-related quality of life (DRQoL) in pediatric patients with an implantable cardioverter defibrillator (ICD) in The Netherlands. Thirty patients were investigated; the mean age was 16.3 years, and the mean duration of implantation was 3.6 years. To assess psychological problems, three domains of the Symptom Checklist (SCL-90-R) were administered to the 25 patients >13 years old. DRQoL was assessed with a disease-specific pediatric questionnaire, the short-form 11-item Worries About (WA)ICDs Scale. Patients ≥13 years old scored significantly higher than the reference group on the domains of anxiety, depression, and sleeping problems of the SCL-90-R (*T* = 7.5, *p* < 0.001; *T* = 5.4, *p* < 0.001; and *T* = 7.8, *p* < 0.001, respectively). Patients who had received an (in)appropriate shock reported more depressive symptoms (*T* = 2.1, *p* < 0.03). Patients with >2 years implant duration (*N* = 19) or who had received an (in)appropriate shock (*N* = 13) showed lower DRQoL scores on the modified WAICD (*T* = 2.1, *p* < 0.04; *T* = 2.1, *p* < 0.5, respectively). Age at implantation or underlying disease did not influence psychological problems or DRQoL. Young ICD patients showed more anxiety, depression, and sleeping disorders. Worries were increased among patients with ICD shocks and in those who had their ICD implanted for >2 years. To determine psychological problems and help children to learn to cope with shocks, proper guidance and monitoring of young ICD patients are recommended.

## Introduction

Treatment with an implantable cardioverter defibrillator (ICD) has significantly improved survival in patients with life-threatening arrhythmias. During the last two decades, ICD therapy has played an increasing role in the management of a diverse population of cardiac patients, such as ischemic and nonischemic cardiomyopathies and inherited proarrhythmic syndromes [4, 11]. Although ICD implantations in children are only 1% of those in adults, outcome data of ICD therapy in children are comparable with adult clinical trial data [2, 28, 29]. However, complication rate, incidence of infection, and frequency of inappropriate ICD shocks appear to be greater in children [6, 16, 17].

With the increasing prevalence of ICD therapy in children, measurement of disease-related quality of life (DRQoL) and psychological assessment have become more essential [14]. Adult studies indicate that ICD treatment can have a negative impact on the DRQoL and psychological functioning of patients [3, 9, 15]. Adult ICD patients show significantly worse psychological and physical functioning [3], and behavioral changes, such as decreased activity, avoidance, depression, and anxiety [13, 18, 21], have been reported. Receiving ICD shocks is a risk factor for psychological distress in adults [23]. However, the adult ICD population differs from the pediatric population in that ischemic heart disease and cardiomyopathy are most prevalent. Furthermore, children have a more active lifestyle compared with most adults [25].

A few studies on quality of life (QoL) in children with an ICD have been published so far [7, 9, 10, 25, 26]. Dubin et al. [9] found difficulties with social interactions in a young ICD population with a mean age of 28 years. DeMaso et al. [7] studied 20 children (median age 15.1 years [range 9–19]) with ICDs. Half of the patients had underlying congenital heart disease. Feelings of anxiety (total and physiological anxiety, social concerns, and worry/oversensitivity) and depression were significantly more prevalent, and their QoL was strongly correlated with these feelings. Feelings of anxiety and depression were also reported by Eicken et al. [10], who studied a group of 16 children (median age 12.2 years [range 4–16]). Seven patients showed signs of generalized depression and/or anxiety. In 3 patients with severe signs of anxiety, ICD shock had occurred within 6 months preceding the investigation. Although studies in adults suggested a negative influence of appropriate or inappropriate shocks on QoL, this has not been confirmed in these pediatric series [26]. Sears et al. [26] described in their recent article QoL to be independent of the occurrence of shocks in pediatric patients. Furthermore, it is unclear if feelings of anxiety and depression are time dependent and may change during longer-term follow-up.

We therefore studied 30 young patients after ICD implantation and analyzed psychological functioning and DRQoL, with special attention paid to the association with shocks and follow-up time.

## Methods

### Patients

The study was approved by the Medical Ethical Committee of the Leiden University Medical Center and adhered to the Declaration of Helsinki. Pediatric patients were recruited from the cardiology departments of four academic medical centers in the Netherlands: Amsterdam Medical Center (11 patients), University Medical Center Groningen (7 patients), University Medical Center Utrecht (2 patients), and Leiden University Medical Center (10 patients). These hospitals have a shared patient database for all ICD patients. Participants were eligible for this study if implantation had been performed before the age of 18 years and if they were able to comprehend Dutch. In total, 35 patients met the enrollment criteria. The treating physicians of the different hospitals asked the patients to participate. Four patients chose not to participate for reasons they did not share. One patient had to be excluded because too many data were missing. The participation rate was 85%.

During a period of 4 months, 30 patients were recruited into the study. First, they received written information about the study and were asked to return the informed consent form. Consent was given by parents for children <12 years old; Consent was given by both patient and parents for children 12–18 years old. Next, one researcher interviewed all children at home or, in four cases because of practical reasons (i.e*.*, distance to the hospital), in the hospital. Information was obtained from the parents about age, sex, number of hospitalizations, parental marital status, and parental educational level. Patients filled in questionnaires in another room than their parents, which took approximately 30–35 min.

### Measures

Patient characteristics included age, weight, and length at the time of ICD implantation, use of antiarrhythmic medication, underlying cardiovascular disease, ICD characteristics, and the occurrence of appropriate or inappropriate shocks. Both chart review and device interrogation were used to assess the presence of shocks.

To assess psychological problems, the SCL-90-R [1, 19] was used. This self-report rating scale consists of 90 items divided into 9 subscales. The SCL-90-R was administered to the participants >13 years old (*N* = 25). The Dutch norm group consists of 2366 randomly selected persons from the general population. For this study, the SCL-90-R subscales anxiety (7 items), depressive symptoms (13 items), and sleeping problems (3 items) were used. Respondents report, on a scale ranging from 1 (not at all) to 5 (very often), whether the described symptom had occurred in the previous week. A greater score represents more symptoms of anxiety, depression, or sleep problems. Cronbach’s α for the 3 subscales in this study group ranged from 0.84 to 0.89, which was in line with the Dutch norm group [1].

ICD is a very specific treatment, so we aimed to use a disease-specific measure to assess DRQoL in this group: the WAICD. This instrument is an adaptation of the 26-item Index of Subjective Concerns for People with ICDs (ISCP-ICD) [30] for use with pediatric ICD patients (DeMaso et al. [7]). Items are scored on a 5-point scale ranging form 0 (not at all true) to 4 (extremely true). Items 7, 20, and 26 are reverse-scored to reflect the opposite valence of these questions. Total WAICD score is determined by the total of items with a scoring range of 0 (no worry at all) to 104 (extremely worried). Greater WAICD scores indicate more worries. The internal consistency of the WAICD (26 items) in our study was adequate (Cronbach’s α 0.77). However, a number of items in our study showed a very low (e.g*.*, items 8, 9, 12, 19, 26) or even negative (item 20) item–total correlation or lack of variance [20]. Therefore, the WAICD Short Form (11-items) was constructed (Cronbach’s α 0.83) and used for further analysis with permission of the original investigator (see Table 1). The two forms (WAICD 26 and 11-item version) correlated well (Pearson’s correlation coefficient [PCC] = 0.086) [20].

**Table 1:** WAICD-SF: 11 items, item–total correlations, and responses

WAICD-SF items (*N* = 30)	WAICD item no. 26	Item–total correlation	Not at all true	A little true	Sort of true	Quite a bit true	Extremely true
1. It bothers me not knowing when the ICD will fire.	1	0.45	12	11	2	1	3
2. I worry about the ICD firing and creating a scene.	2	0.49	18	3	3	3	2
3. I am afraid of being alone if the ICD fires and I need help.	3	0.63	10	9	3	2	5
4. I worry about the ICD not firing sometime when I need it.	4	0.37	18	7	3	–	1
5. I can’t stop thinking about the ICD.	6	0.42	16	6	6	1	–
6. I worry that the ICD will fire when it’s not supposed to.	14	0.56	17	6	3	–	3
7. I worry about how I will feel when the ICD fires.	15	0.66	12	7	5	2	3
8. I am nervous that if I exercise my heart might start beating faster and make the ICD fire.	16	0.54	13	7	3	3	3
9. I worry about not being able to get a job in future because of the ICD.	19	0.38	21	3	5	–	–
10. I worry about traveling to a place where there aren’t any doctors who know about ICDs.	23	0.42	17	9	1	–	2
11. I worry that paramedics don’t know how the ICD works.	24	0.59	17	7	3	1	1

*SF* short form

### Statistical Analyses

Data were analyzed with the use of SPSS 17.0 software (Chicago, IL). Mean scores (M) of anxiety, depression, and sleeping problems in children with an ICD were compared with the instrument norms using Student *t* test. Scores on the WAICD-SF were compared with scores of the original instrument. Pearson and Spearman correlations were calculated between DRQoL, psychological problems, time since implantation, and having had shocks or not. Especially this last variable has been identified as possible determinants of well-being in ICD patients [7, 10, 21]. Time since implantation was a variable added by the researchers of the current study. χ^2^ tests were performed to test the relationship between categorical data and DRQoL and psychological problems. PCCs were calculated to measure relations between the WAICD-SF and the SCL-90-R. For all calculations, *p* ≤ 0.05 was considered significant. Results were compared with age- and sex-matched reference groups where possible.

## Results

### Study Subjects

Mean age at time of assessment was 16.3 years (range 9–23) with a mean duration since ICD implantation of 3.6 years (range 1–9). Only seven children were <13 years old at the time of assessment. Table 2 lists the demographic and illness-specific characteristics of the participants. No differences were found between the children interviewed at home or those interviewed in the hospital regarding demographic or disease-related variables and study outcomes. Twelve participants had been admitted to hospital during follow-up. Five underwent battery replacement, 4 underwent lead replacement, and 3 were monitored because of multiple shocks. No differences were found between hospitalized and nonhospitalized patients. Nineteen children were taking beta-blockers. Fifteen patients received ≥1 shock: 10 patients received an appropriate shock, and 7 patients received an inappropriate shock. Lifestyle limitations for this group were to avoid competitive sports (both before and after implantation) and to avoid contact sports after implantation.

**Table 2:** Sample characteristics and cardiac illness description in 30 pediatric patients with an ICD

No. of patients included	30
Age at implant (years)	13.7 ± 3.6^a^ (range 4.8–22.4)
Sex (male/female)	15/15
Weight (kg) at implant	48.4 ± 17.0 (range 15–94)
Height (cm) at implant	160.3 ± 19.5 (range 105–190)
Beta-blockers used (no.)	19
ICD indication	
SCD survivor	13
Primary prevention	17
Cardiovascular disease	
Cardiomyopathy	6
Primary electrical disease	17
Congenital	7
Implant characteristics	
Epicardial/transvenous approach	2/28
Single/dual chamber	26/4
Generator abdominal/subpectoral	4/26
DFT at implant (mean)	15.1 ± 5.7
Follow-up	
Follow-up after implantation (y)	3.6 ± 2.6

*SCD* sudden cardiac death, *DFT* defibrillation threshold

^a^Plus–minus values are expressed as mean ± SD

At the time of evaluation, 25 children were living with both parents, and 5 children were living with one of their parents. Seven children were in primary school (ages ≤12 years); 19 patients were in secondary school (ages 12–19 years); and 4 patients were working >20 h per week (ages >19 years).

### DRQoL of Pediatric Patients with an ICD

Results of the WAICD-SF showed that mean total scores were comparable with the norm group in the original study by DeMaso et al. [7] (*M* = 29.5 vs. 37.2, respectively). The main worries of the respondents in our study group reported on the WAICD-SF (see Table 3) were as follows: “I am afraid of being alone if the ICD fires and I need help” (66%) followed by “I worry about how I will feel when the ICD fires” (59%) and “I am nervous that if I exercise my heart might start beating faster and make the ICD fire” (55%). Nearly 40% of the pediatric patients were a little concerned about not knowing when the ICD would fire.

**Table 3:** Psychological problems reported on three SCL-90-R subscales

Groups	Mean (SD) depressive symptoms (*N* = 16 items [range 16–80])	Mean (SD) anxiety (*N* = 10 items [range 10–50])	Mean (SD) sleep problems (*N* = 3 items [range 3–15])
ICD sample (*N* = 23)	31.6* (6.2)	17.3* (3.9)	7.6* (1.9)
Reference group (*N* = 2366)^a^	21.6 (7.6)	12.8 (4.4)	4.5 (2.2)

* *p* < 0.001

^a^Dutch reference group of the SCL-90-R: characteristics of reference group [20]: 50% female, mean age = 41.1, SD = 14.5, age range 17–88 years

### Psychological Problems of Pediatric Patients with an ICD

Scores of the patients with an ICD who were ≥13 years old on the domains of anxiety, depression, and sleeping problems of the SCL-90-R were significantly greater than the reference group (*T* = 7.5, *p* < 0.001; *T* = 5.4, *p* < 0.001; and *T* = 7.8, *p* < 0.001, respectively) (see Table 3). No sex differences were found. Patients who had received one or more shocks (both appropriate and inappropriate) reported more depressive symptoms (*T* = 2.1, *p* < 0.03). There were no differences between the patients with underlying primary electrical disease, cardiomyopathy, or congenital heart disease, although the number of patients in the respective subgroups were relatively low, thus causing a lack of power.

### Correlates of DRQOL

Pediatric patients with an implantation duration >2 years (*N* = 19) showed significantly more worries on the WAICD-SF (*T* = 2.1, *p* < 0.04). Patients who had not yet received a shock (both appropriate and inappropriate) (*N* = 15) worried less than the other patients (*T* = 2.1, *p* < 0.05) (see Table 4). Age at implantation and sex were not associated with worrying or ICD-related HRQoL. There were no differences between the patients with underlying primary electrical disease, cardiomyopathy, or congenital heart disease. Because more than half of the patients (*N* = 17) had a primary electrical disease and therefore had no restrictions outside of their ICD, no correlation could be found between severity of illness and QoL.

**Table 4:** Mean (SD) differences between time since implantation and number of shocks and DRQoL (WAICD-SF)

Variables	WAICD-SF mean (SD) *p*	
Time since implantation		
<2 years (*N* = 11)	5.6 (4.3)	
>2 years (*N* = 19)	11.9 (8.7)	0.049*
Number of shocks		
0 (*N* = 15)	7.2 (5.3)	
≥1 (*N* = 15)	12.9 (9.6)	0.045*

*SF* short form

* *p* < 0.05

## Discussion

In this multicenter study, we investigated psychological functioning and DRQoL in pediatric patients with an ICD. A significantly increased rate of anxiety, depression, and sleep problems, as well as worries about their ICD, were reported. These results are in line with previous research with pediatric patients [7]. We found that length of implantation (>2 years) was associated with more worries. The number of shocks, both appropriate and inappropriate, also showed an important impact on worries in our pediatric ICD population. Patients who had received one or more shocks reported significantly more worries compared with those who had not received a shock. This finding is consistent with previous research showing that the unpredictability and frequency of ICD shock delivery [6, 10, 15] are associated with negative emotions.

Studies evaluating QoL in adults with an ICD found greater depression and anxiety levels compared with the general population [13–15]. However, it seems that the survival of an out-of-hospital cardiac arrest [14], persistence of ventricular arrhythmias [3], or severity of the underlying disease [13] is more important than ICD implantation itself. Studies in young ICD patients show more signs of depression and anxiety as well [7, 10]. However, these studies did not differentiate between the effect of the underlying disease and the ICD implantation.

Cardiac problems have a major (physical) impact on general HRQoL. For example, in the study by Sears et al. [26], pediatric ICD patients (*N* = 60) reported on the PedsQL significantly lower psychosocial and physical HRQoL than healthy peers and lower physical HRQoL than chronically ill samples. Moreover, female ICD patients (*N* = 25) reported lower psychosocial, physical, and cardiac-specific HRQoL than male patients.

In our study, more than half of the patients had a primary electrical disease and were therefore a relatively healthy population. A previous study showed that children who were carrier for an inherited cardiovascular disease, including long QT syndrome, did not show differences in QoL compared with their healthy peers [27]. However, ICD implantation in children with primary electrical disease might have a significant impact on their QoL [22]. These studies make it more likely that ICD implantation has a much greater influence than the presence of the primary electrical disease itself. In the present study, we found a significant effect of the occurrence of shocks on QoL. This has not been found previously in young patients [26], whereas adult studies showed conflicting results: Some of them showed an influence of shocks [13, 15, 18], whereas others did not [21]. The effect of duration of ICD therapy on worrying has neither been found previously. This finding shows that pediatric patients do not necessarily accommodate well to their ICD (more than four of five pediatric patients avoided places or activities after ICD implantation [26] (female patients avoided places significantly more), and they probably need psychological support not only before and immediately after ICD implantation but also during long-term follow-up [5, 8, 25].

### Limitations

However, different limitations should be given consideration. One of the major restrictions of this study is the small sample size, which makes statistical analyses difficult to conduct and interpret (low statistical power), a common problem in this illness group [10, 16]. Furthermore, the large range of duration of ICD therapy, the heterogeneity of the underlying diseases, and the variety of the age of the patients also cause problems in the generalization of the results. Because most of these young patients led a relatively normal life besides the presence of their ICD, no correlation could be found between severity of illness and QoL. Another limitation is the fact that the age range of the reference group of the SCL-90-R (see Table 3) was not congruent with the ages of the ICD patients under study. Limited SCL-90-R data are available from pediatric patients with a chronic disease or handicap. In one study [12], SCL-90-R data of 17 patients with pectus excavatum (13 male and 4 female, *M* 19.6 years, SD 2.5 years at the second time [4 years later] of investigation) were within the normal range. A fourth limitation is the fact that a newly developed disease specific measure (WAICD) had to be adapted because of insufficient psychometric properties in our population under study. Its value as a DRQoL assessment tool must be proven in clinical practice, in conjunction with other more widely used measures, to provide a better understanding of the impact of ICD treatment in children.

## Conclusion

The present study shows that young ICD patients experience psychological- and disease-related consequences of their treatment (e.g*.*, do not feel safe, tire easily, and worry about needing an ICD). Compared with normal controls, ICD patients showed more anxiety, depression, and sleeping disorders. Depression level was increased among patients with ICD shocks. In addition, worries about their ICD were increased in patients with ICD shocks and in those who had their ICD for >2 years. These findings underscore the necessity of proper guidance and monitoring of ICD patients to cope with the shocks and hence determine possible psychological problems [24]. Patients with frequent shocks and longer ICD treatment are especially at risk. Last, these findings support the necessity to decrease both appropriate and inappropriate shocks to the lowest possible incidence.
